# Hyperspectral dimension reduction and navel orange surface disease defect classification using independent component analysis-genetic algorithm

**DOI:** 10.3389/fnut.2022.993737

**Published:** 2022-10-19

**Authors:** Jing Li, Liang He, Muhua Liu, Jinyin Chen, Long Xue

**Affiliations:** ^1^Jiangxi Key Laboratory of Modern Agricultural Equipment, College of Engineering, Jiangxi Agricultural University, Nanchang, China; ^2^Collaborative Innovation Center of Postharvest Key Technology and Quality Safety of Fruits and Vegetables in Jiangxi Province, Nanchang, China; ^3^Key Laboratory of Optics-Electrics Application of Biomaterials of Jiangxi Province, Jiangxi Agricultural University, Nanchang, China

**Keywords:** canker, penicilliosis, navel orange, hyperspectral image, ICA-GA

## Abstract

Canker is a common disease of navel oranges that is visible before harvest, and penicilliosis is a common disease occurring after harvest and storage. In this research, the typical fruit surface, canker spots, penicillium spore, and hypha of navel oranges were, respectively, identified by hyperspectral imaging. First, the light intensity on the edge of samples in hyperspectral images was improved by spherical correction. Then, independent component images and weight coefficients were obtained using independent component analysis. This approach, combined with use of a genetic algorithm, was used to select six characteristic wavelengths. The method achieved dimension reduction of hyperspectral data, and the testing time was reduced from 46.21 to 1.26 s for a self-developed online detection system. Finally, a deep learning neural network model was established, and the four kinds of surface pixels were identified accurately.

## Introduction

Navel orange is an important fruit crop grown in Jiangxi Province, China and many other agricultural areas in subtropical regions. The fruit is generally round, oblate, or elliptic in shape and usually orange-yellow or orange-red in color (1). The calyx of the fruit has a few immature pericardial groups, which form the characteristic navel, and the core is solid or semi-solid. In the process of transportation and packaging of navel oranges after harvest, a spore from a single navel orange can cause an entire batch to start sporing within a short time (2). Therefore, the post-harvest sorting of navel orange is conducive to improving their value and increasing the preservation period, and technology to accomplish this objective is urgently needed for fruit production (3).

In present-day fruit sorting operations, machine vision technology is mainly used to assess fruit appearance quality, including fruit shape and visible defects, for example, brown spots, spores, granulation, edema, etc. However, for some defects, such as decay caused by fungi, machine vision methods seem ineffective. The main reason is that the skin color of a decayed area can be close to that of the normal skin areas, so it is hard to distinguished using images from a color camera. However, the skin damage is related to the characteristics of the fruit skin that can be detected by near infrared spectroscopy (NIRS). Moreover, NIRS can also be used for non-destructive testing of the internal components of fruits, such as soluble solids (4), acidity, and other traits (5, 6). Although NIRS technology can reflect the internal quality of fruit, only a small part of the fruit surface can be collected, and the fruit appearance quality cannot be perfectly determined.

Hyperspectral image technology combines the advantages of spectrum- and image-based approaches (7). It not only can achieve fruit appearance image inspection, but can also collect the surface spectra which can be used to detect the invisible defect (8, 9). The hyperspectral image is a three-dimensional image. Through the selection of characteristic wavelengths of hyperspectral images, simultaneous external and internal fruit quality testing can be realized; this is a major development trend in the field of fruit detection (10). Hyperspectral image technology can be used to detect meat quality (11), orange spores, cucumber frostbite (12), guava maturity (13), strawberry ripeness (14), Moisture of Okra (15), and many other food defects (16–18). However, hyperspectral images contain large amounts of wavelength information, thus requiring a long time to collect information; accordingly, its online detection applications are limited. Therefore, the study of hyperspectral data dimension reduction technology has become the key to improving the speed of online detection. Current dimension reduction methods mainly include genetic algorithm (19), principal component analysis (20, 21), independent component analysis (22), and some deep learning (23) methods. However, most approaches have only been applied to hyperspectral test platforms, and online detection has not yet been realized.

In this study, the hyperspectral images of navel orange samples were dynamically collected using a self-designed online detection system. The spectra ranged from 975.18 to 2,196.2 nm. The method employed combined independent component analysis (ICA) with genetic algorithm (GA) to select the characteristic spectra. The dimensions of the hyperspectral images were reduced, and then the cankered and spored fruit were separated. This study had the following specific objectives: (1) spectral preprocessing of the collected hyperspectral images; (2) use of an ICA method to obtain independent component images and weight coefficients; (3) combining these data with GA after characteristic selection of wavelengths to achieve dimensionality reduction of hyperspectral data; (4) establishment of a deep neural network method of classified of navel orange surface defects and subsequent automated classification of fruits with a normal versus defective surface. Six characteristic wavelengths were selected by ICA-GA, and the surface defect detection model of navel orange was established based on LSTM. Finally, the online detection of navel orange was achieved.

## Materials and methods

### Experimental materials

**FIGURE 1:**
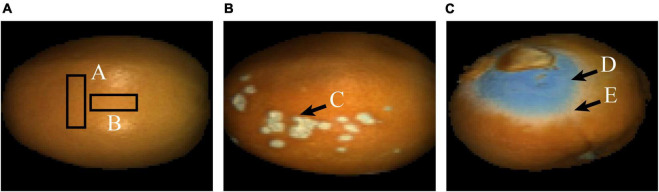
False-color images of the typical navel orange. **(A)** Navel orange sample with an intact surface; **(B)** navel orange sample with canker; **(C)** navel orange sample with penicilliosis.

The navel orange samples used in this experiment were collected in November 2019 from Ganzhou City, Jiangxi Province, China. After harvest, the navel oranges were first cleaned in the laboratory. Thirty normal navel orange samples without defects (group 1) and thirty navel oranges with canker defects on their surfaces (group 2) were selected. Then, the hyperspectral image of the samples in categories 1 and 2 were acquired. Meantime, one navel orange inoculated with penicilliosis was stored with some normal navel oranges in the laboratory. After 30 days, 30 infected navel orange were selected as samples with penicilliosis on their surfaces (group 3), and the hyperspectral image data of them were also acquired. Images of samples from each category are shown in Figures 1A–C, respectively.

In order to distinguish navel oranges with surface defects online, it is necessary to identify the above defects on surfaces of navel orange samples. In this study, navel orange surfaces were mainly divided into four categories: normal navel orange surface without defects, cankered surface, penicillium spore surface, and hypha surface. Figure 1A shows a navel orange without defects on its surface, and the category is defined as Surface. Since normal navel oranges have a large surface range, only rectangular areas A and B were selected. Figure 1B shows the surface of a navel orange with cankers, as indicated by the arrow in area C. This type of sample is referred to as Canker. Figure 1C is the image of a penicilliosis navel orange sample. Areas D and E were, respectively, the surface of penicillium spores and penicillium hypha, which are referred to as Spore and Hypha, respectively.

### Hyperspectral imaging sorting system

**FIGURE 2:**
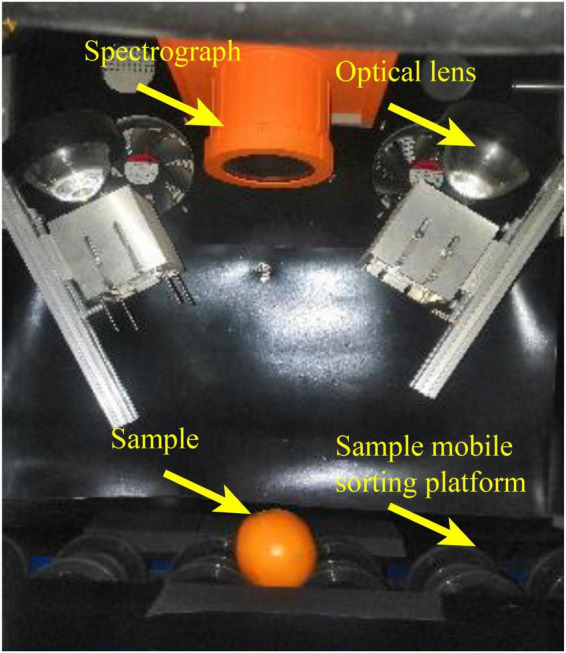
Hyperspectral imaging sorting system.

The hyperspectral image system mainly includes a hyperspectral camera (SWIR-CL-400-N25E, SPECIM, Finland), two 150-Watt halogen lamps, two focusing lenses, and a mobile sorting platform setup, as show in Figure 2. Before collecting hyperspectral images, the lamp was turned on for about 15 min, and the white and black calibration images of the hyperspectral camera were captured. The white calibration image was acquired from a 99.9% reflectance white board (Spectralon SRT-99-100, Labsphere Inc., North Sutton, NH, USA) 30 cm below the camera. The black calibration image was acquired when the lens was completely shielded.

The hyperspectral images of samples were corrected using Equation (1):


(1) $$I=\frac{I_{d\,a\,t\,a}-I_{b\,l\,a\,c\,k}}{I_{w\,h\,i\,t\,e}-I_{b\,l\,a\,c\,k}}$$

Here, *I*_*data*_ represents the acquired hyperspectral image, *I*_*black*_ represents the black calibration image, *I*_*white*_ represents the white calibration image, and *I* represents the corrected image. The exposure time and the frame rate of the hyperspectral camera were 1.8 ms and 120 f/s, respectively.

### Spectral preprocessing

**FIGURE 3:**
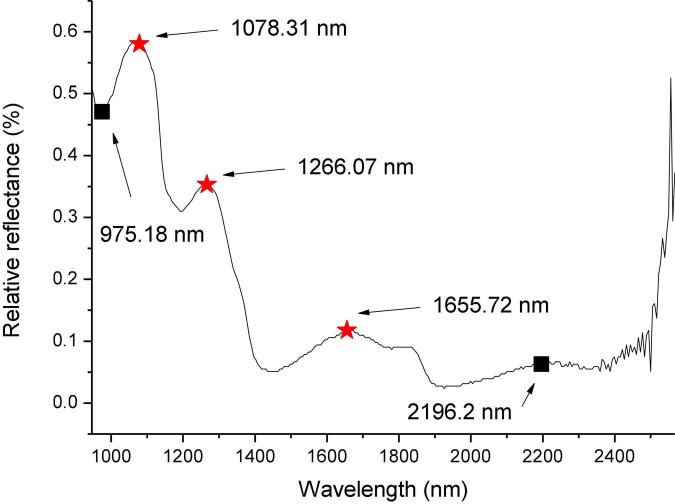
Reflection spectrum of a normal navel orange surface.

The average spectrum of rectangular areas A (in Figure 1) corrected by the black and white calibration images is shown in Figure 3. It can be seen that there is considerable noise at both ends of the spectrum, at ranges of 946.43–975.18 nm and 2,196.2–2,256.82 nm, respectively. Therefore, in the subsequent processing, these two parts of spectra were eliminated, and the spectral range within 975.18–2,196.2 nm between the two black rectangular boxes in Figure 3 was used. The red asterisk in Figure 3 represents the three wave peaks of the surface spectral curve of navel oranges, which are respectively, 1,078.31, 1,266.07, and 1,655.72 nm. The hyperspectral images corresponding to these three bands were selected to produce false color images of navel oranges, like the one shown in Figure 1.

As navel oranges are spherical fruits, the reflected light on the upper surface of the navel oranges collected by the hyperspectral camera is the strongest. In contrast, the reflected light at the edges of the navel oranges is very weak. In order to improve the reflected light intensity on the surface of the spherical fruits and increase the light intensity at the edges, the hyperspectral images of each spherical fruit were corrected (24). This is achieved through the following correction process: (1) the binary-value image of the sample is built using a 1,078.31 nm wavelength; (2) the center of gravity of the binarization region is found, and the image is then segmented into 16 equal parts; (3) the height of the sample based on the distance from the center of gravity to points in each of the 16 equal parts is calculated; (4) the height of each pixel in the segmented binary image according to four quadrants is calculated, and the reflected light intensity is then corrected. Then, standard normal variate (SNV) transformation is used to eliminate baseline drift.

### Establishment of deep neural network model

Deep neural networks are widely used in deep learning and image recognition (25–27). In this study, a long short-term memory (LSTM) deep neural network was applied to 217 spectra in the range of 975.18–1,146.75 nm to establish a classification model. Before establishing a LSTM deep neural network, training and validation datasets for training model should be provided. From Figure 3, it can be seen the reflection is the strongest at a wavelength of 1,078.31 nm. Based on the 1,078.31 nm hyperspectral images, the tool “Image Labeler” in MATLAB (MathWorks Ltd., Natick, MA, USA) was used to select the most representative pixels on the surface of navel oranges from the prediction dataset. For this dataset, 15 penicilliosis navel oranges, six rotten navel oranges, 30 cankered navel oranges, and 16 normal navel oranges were selected. After sample selection, the image “label” and file “gTruth” were generated for each navel orange. The image “label” contains the features selected from each navel orange. The file “gTruth” contains the file storage location of the corresponding file and the storage location and classification name of the corresponding image “label.”

Thus, 10,405 data points were obtained for training. As shown in Table 1, 1,300 data points for each category were randomly selected as the modeling data, thus obtaining a total of 5,200 modeling data points. These data were randomly divided into a training dataset, validation dataset, and test dataset according to the approximate ratio of 0.8:0.1:0.1.

**TABLE 1:** Spectra number of each category.

Category	Spectra number	Selected spectra number	Spectra number of training dataset	Spectra number of validation dataset	Spectra number of test dataset
Spore	1,954	1,300	1,065	123	112
Hypha	1,374	1,300	1,044	135	121
Canker	3,302	1,300	1,016	161	123
Surface	3,775	1,300	1,035	121	144
Total	10,405	5,200	4,160	540	500

The LSTM model is mainly composed of five layers: (1) sequenceInputLayer, (2) bilstmLayer, (3) fullyConnectedLayer, (4) softmaxLayer, and (5) classificationLayer. The second layer has 200 hidden layer nodes, and the fifth layer has four output categories. The numbers of hidden layer nodes are selected by comparing the results using 50, 100, 150, 200, and 250 hidden layer nodes. The LSTM cell with a Forget Gate can be mathematically expressed as follows:


(2) $$f_{t}=\sigma \,\left(W_{f\,h}\,h_{t-1}+W_{f\,x}\,x_{t}+b_{f}\right),$$

$$i_{t}=\sigma \,\left(W_{i\,h}\,h_{t-1}+W_{i\,x}\,x_{t}+b_{i}\right),$$

$${\tilde{c}}_{t}=\tanh\left(W_{\tilde{c}\,h}\,h_{t-1}+W_{\tilde{c}\,x}\,x_{t}+b_{\tilde{c}}\right),$$

$$c_{t}=f_{t}\cdot c_{t-1}+i_{t}\cdot {\tilde{c}}_{t},$$

$$o_{t}=\sigma \,\left(W_{o\,h}\,h_{t-1}+W_{o\,x}\,x_{t}+b_{o}\right),$$

$$h_{t}=o_{t}\cdot \tanh\left(c_{t}\right)$$

Where *f_t_* is the forget gate, and the value of *f_t_* can decide what information will be thrown away from the cell state. Where *c_t_* denotes the cell state of LSTM. _*W_i_*_, $W_{\tilde{c}}$, and _*W_o_*_ are the weights.

### Selection of characteristic wavelengths

The data collected by the hyperspectral imaging system are usually very large. Therefore, a method of model establishment that uses a small number of characteristic wavelengths cannot only reduce the time spent in modeling, but also effectively avoid the problem of information similarity between adjacent wavelengths while reducing the over-fitting of the model. Therefore, using characteristic wavelengths to establish prediction models can reduce data calculation and meet the efficiency requirements of online detection.

The extraction methods of characteristic wavelengths include principal component analysis (PCA) and independent principal component analysis (ICA). ICA has a superior applicability to solve the problem of blind source separation in spectral analysis (28). Accordingly, this study adopted the ICA method to extract characteristic spectra. The main steps are as follows. First, the binarization extraction of the hyperspectral image is conducted to obtain a mask, which is used to separate the background and the fruit area. According to the position of fruit pixels in the mask, the hyperspectral image is subjected to two-dimensional decomposition, thus obtaining a two-dimensional array. The array included the spectra of the fruit surface, but not the background part in the mask. Then, the first six independent components and the corresponding weight coefficients were obtained by ICA analysis of spectral data. Finally, six independent components were reconstructed according to the positions of the mask pixel points, and the values of independent components. Then the characteristic wavelengths can be selected by the ICA analysis based on the corresponding weight coefficients.

### Genetic algorithm

For online detection, the number of characteristic wavelengths should be reduced as much as possible. Therefore, on the basis of the selected wavelengths using ICA analysis, a genetic algorithm (GA) was applied to select characteristic wavelengths once more (29, 30). A genetic algorithm is a kind of efficient global search optimization algorithm that can find the optimal solution within a large solution space, and its global search ability can greatly improve search efficiency while avoiding local minimal solutions. In this paper, the main genetic algorithm program was written using the software MATLAB. The characteristic wavelengths were selected by GA based on the accuracy of the test dataset as the fitness function. The following main parameters were used for the genetic algorithm: six characteristic wavelengths were selected without repetition each time. That is, the number of individuals was six, the population contained six individuals, the maximum genetic algebra was 30, and the generation gap was 0.9.

## Results and discussion

### Spherical fruit correction

**FIGURE 4:**
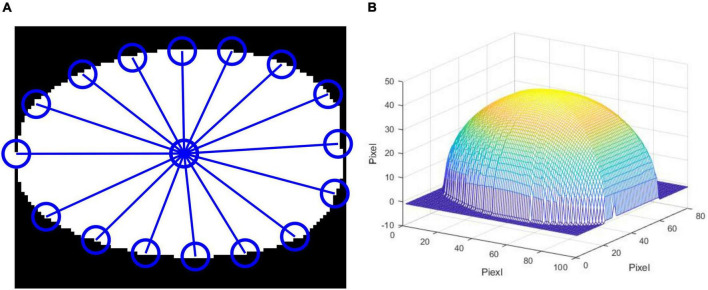
Correction of the spherical fruit. **(A)** segmentation of the mask image, **(B)** 3D reconstruction of spherical fruit.

The segmented and reconstructed navel orange 3D image is shown in Figure 4.

**FIGURE 5:**
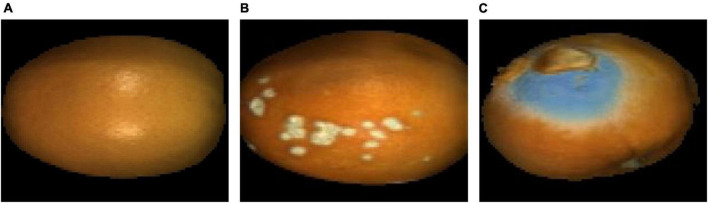
False-color images after spherical correction. **(A)** Navel orange sample with an intact surface; **(B)** navel orange sample with cankers; **(C)** navel orange sample with penicilliosis.

For the corrected hyperspectral images, the 1,078.31, 1,266.07, and 1,655.72 nm wavelength images were extracted and fused into false-color images, as shown in Figure 5. Compared with Figure 1, the light intensity of the edge was significantly improved.

**FIGURE 6:**
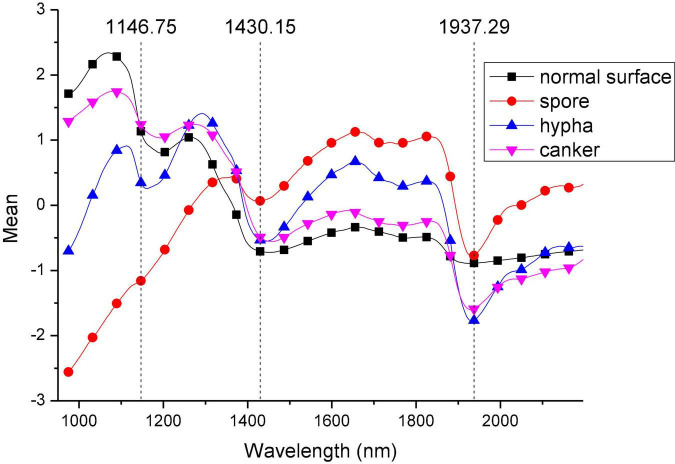
Spectral curves of the four categories of surface after spherical correction and standard normal variable (SNV) transformation.

After spherical correction, the hyperspectral images of navel oranges were decomposed into two dimensions, and the spectra were preprocessed using standard normal variate (SNV) transformation to eliminate baseline drift. Corrected characteristic spectra of normal Surface, Canker, Spore, and Hypha samples are shown in Figure 6. In the wavelength range of 975.18–1,146.75 nm, the spectral value of Surface is the highest, while the spectral value of Spore is the lowest. In the wavelength range of 1,146.75–1,430.15 nm, the spectral curves of Canker, Spore, and Hypha show some differences. At the wavelength of 1,430.15 nm and 1,937.29 nm, the spectral values of Surface and Hypha were the lowest. The four types of spectra all had a double-peak structure, and at 1,430.15 nm, there was a significant trough. At 1,937.29–2,196.26 nm, the spectral curves of Canker, Spore, and Hypha samples all increased, while the Surface spectral curve was relatively stable.

### Model using full bands

The LSTM01 model was obtained by using the whole spectra, which included 217 bands. After training, the accuracy of the training, validation, and test dataset were 91.80, 90.56, and 92.81%, respectively. The modeling time was about 3,265.691 s, and the average test time for each orange was about 46.21 s.

### Characteristic wavelengths using independent component analysis

**FIGURE 7:**
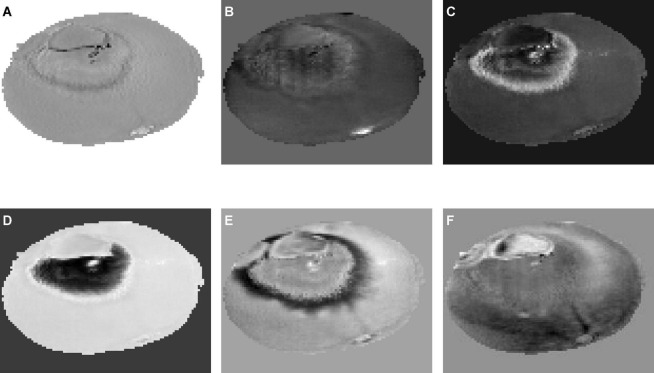
Six independent component images by independent component analysis. **(A)** ICA01, **(B)** ICA02, **(C)** ICA03, **(D)** ICA04, **(E)** ICA05, **(F)** ICA06.

Six independent component images were obtained, as shown in Figure 7. ICA01–ICA06 represent the first through sixth independent component images, respectively. Figure 7 shows the ICA result of a navel orange sample with penicilliosis. It can be seen that ICA03, ICA04, and ICA05 are sensitive to penicilliosis navel orange images. ICA03 and ICA05 are sensitive to penicillium hypha images, and ICA04 is sensitive to penicillium spore category images.

**FIGURE 8:**
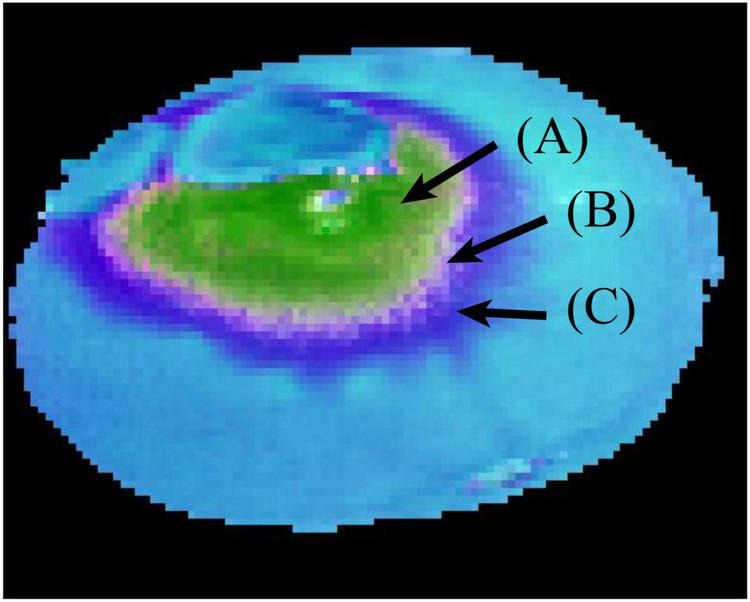
Pseudo-color image of a penicilliosis navel orange sample.

Figure 8 is a pseudo-color image of a penicilliosis navel orange sample synthesized by ICA03, ICA04, and ICA05. The figure shows that the spore region (A) and hypha region (C) are relatively obvious, and the region (B) between regions (A) and (C) is the transitional region between spores and hyphae. On this basis, the characteristic wavelength can be selected through the weight coefficients of the wavelength variates of the three independent components.

**FIGURE 9:**
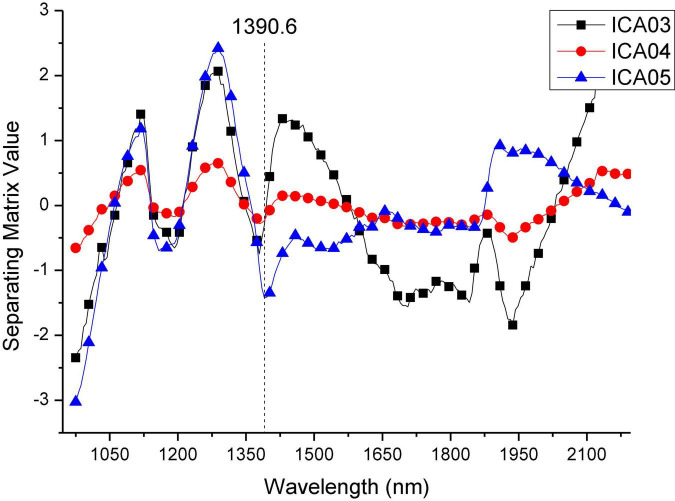
Separating matrix of ICA03, ICA04, ICA05.

**FIGURE 10:**
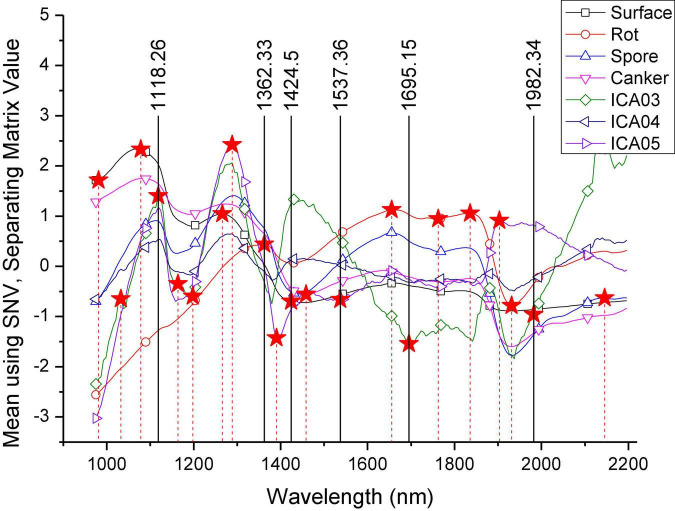
Selection of characteristic wavelengths.

Figure 9 shows the separating matrix values of the wavelengths for independent components ICA03, ICA04, and ICA05. It can be seen that ICA03 and ICA05 have similar curve trends in the range of 975.18–1,390.6 nm. The curves both contain two wave peaks. In the range of 1,390.6–2,196.26 nm, the three independent component curves showed obvious differences. According to the curve trend of the independent component, the characteristic wavelengths were selected based on the peaks and troughs of the curves. In the selection process, in order to contain as much information about the classified features as possible, the selection of feature wavelengths also included the peaks and troughs of the spectral curves of the four categories in Figure 6. Thus, a total of 21 characteristic wavelengths were selected, which are marked with asterisks in Figure 10 and Table 2.

**TABLE 2:** List of 21 characteristic wavelengths.

Number of wavelengths	Characteristic wavelengths (nm)
21	980.930, 1,032.55, 1,078.31, *1,118.26*, 1,163.83, 1,197.95, 1,266.07, 1,288.74, *1,362.33*, 1,390.60, *1,424.50*, 1,458.38, *1,537.36*, 1,655.72, *1,695.15*, 1,762.73, 1,835.94, 1,903.51, 1,931.66, *1,982.34*, 2,145.61

Italic values are the result of wavelength selection.

The LSTM02 model was obtained by training the LSTM model with 21 characteristic wavelengths. The accuracies of the training dataset, validation dataset, and test dataset were 94.87, 94.07, and 95.01%, respectively. The modeling time was about 328.4015 s, and the average test time for each orange was about 3.71 s. Compared with the LSTM01 model trained with full spectra, it can be seen that the accuracy has been improved, the modeling time has been reduced by about 10 times, and the number of wavelengths used has been reduced from 217 to 21.

### Characteristic wavelengths using genetic algorithm

Based on the 21 characteristic wavelengths selected by ICA, GA was used for further dimension reduction, and the six characteristic wavelengths are 1,118.26, 1,362.33, 1,424.50, 1,537.36, 1,695.15, and 1,982.34 nm. In Table 2, the values specified in italic font are the six characteristic wavelengths. Then, the LSTM03 model was established with six characteristic wavelengths. The accuracies of the modeling set, prediction set, and verification set were 94.03, 93.148, and 93.41%. The modeling time was about 105.223 s, and the average test time for each orange was about 1.26 s. It can be seen that through the selection of wavelengths by GA, the number of wavelengths is reduced from 21 to 6, and the time required for modeling is also reduced by about three-fold compared with the LSTM02 model.

### Prediction results

The prediction results show that the prediction accuracy of LSTM01, LSTM02, and LSTM03 models were 92.81, 95.01, and 93.41%, respectively. Although the prediction accuracy of LSTM03 model was slightly lower than that of LSTM02, the prediction time of LSTM03 model for each navel orange was the shortest, only 1.26 s, the prediction time of LSTM02 was 3.7 s, and the prediction time of LSTM01 is the longest, 46.21 s. Thus, in order to ensure the accuracy and efficiency at the same time, the LSTM03 model was better than LSTM02, which is conducive to the online monitoring of fruits by accurately predicting the location of penicillium spore defects.

**FIGURE 11:**
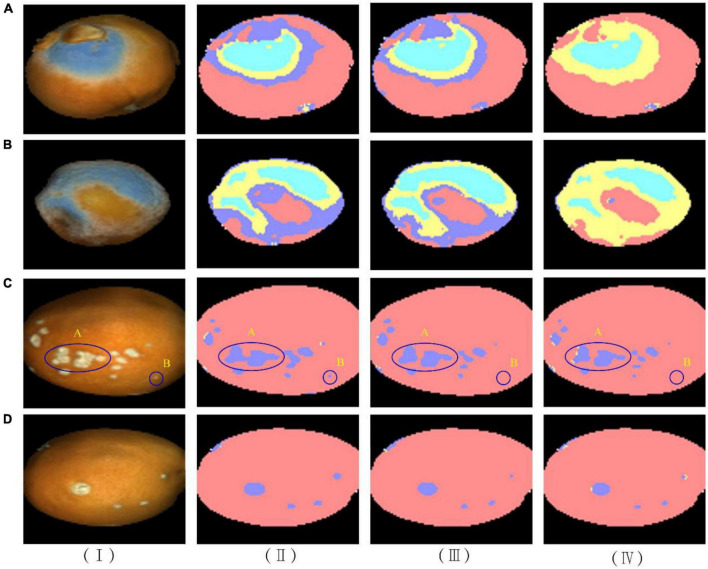
Result of the three LSTM models for four typical samples. (I) Sample hyperspectral images and corresponding images for models (II) LSTM01, (III) LSTM02, (IV) LSTM03.

Figure 11 shows the classification results of four navel oranges in the application of the three LSTM models. In the column (I), (A) and (B) are pseudo-color images of penicilliosis navel oranges, while (C) and (D) are pseudo-color images of navel oranges with canker. The proportions and positions of penicillium disease and canker on the navel orange surface differ. In the column (II), the prediction results of the LSTM01 model are shown. It can be seen that part of the hypha area was misjudged as canker in (A) and (B), but the location of penicillium disease was fairly accurate. The predictions for (C) and (D) were relatively accurate. A smaller canker part was detected in circular region B. However, two canker regions were connected in elliptic region A. In the column (III), the prediction results of the LSTM02 model are shown; (A) and (B) show that the prediction results of penicilliosis were good, but some hypha area were still misjudged as canker. The canker locations in (C) and (D) also had high recognition rates. Compared with the predicted results of the LSTM01 model, two canker parts in the elliptic region A were identified separately, while the smaller canker was not successfully identified and was instead misjudged as normal surface, as shown in circular region B. Among the results of LSTM03 in the column (IV), the prediction results showed that hypha areas in (A) and (B) are accurately identified. The discriminant results of canker in (C) and (D) are similar to that of the LSTM02 model, and the overall area of the discriminant result is slightly smaller than that of the LSTM02 model.

**FIGURE 12:**
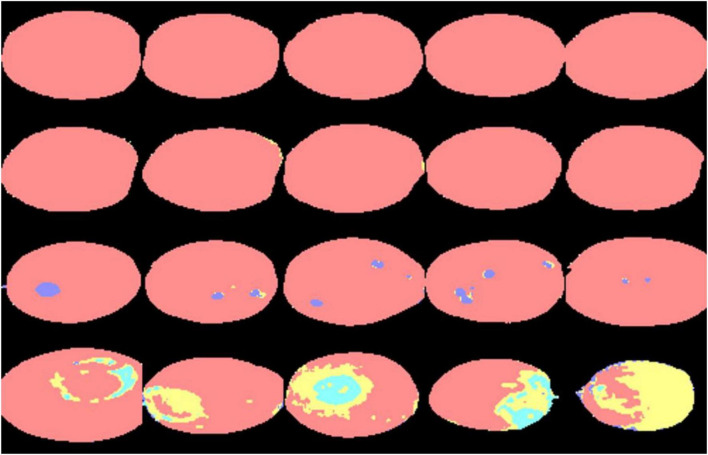
Discrimination results of 20 navel orange samples.

Figure 12 shows the classification results for 20 navel oranges using the LSTM03 model. The first and second rows are the discriminant results of hyperspectral data from navel orange samples collected on the first and fifth days after picking, respectively. It can be seen that the hyperspectral image data collected on the fifth day included misjudged hyphae, especially the edge part of navel oranges. The third row is the discrimination result of hyperspectral image data with canker, which shows that this model can accurately identify the canker location and size on navel orange surfaces. The fourth row shows the discrimination results of hyperspectral image data collected after 30 days in the laboratory. This model can accurately identify penicilliosis on navel oranges.

## Conclusion

Based on ICA and wavelength optimization by GA, a total of six characteristic wavelengths were selected to establish a deep learning neural network model. The model was used to classify and detect surface defects of navel oranges. The categories included penicillium spore, penicillium hypha, canker, and normal navel orange surfaces. The characteristic wavelengths were, respectively, 1,118.26, 1,362.33, 1,424.50, 1,537.36, 1,695.15, and 1,982.34 nm. Through the selection of characteristic wavelengths, the test time of the LSTM03 model for each navel orange sample was reduced from 46.21 s (with the full spectrum model) to 1.26 s. It was conductive to the hyperspectral online detection of fruit, and its prediction accuracy was also improved. It can be seen that this method can be used to detect the surface defects of navel oranges online. However, the hyperspectral images were collected in this experiment through translation movement of navel orange samples. Thus, only images from the upper surface of the navel orange were collected, while lower surface images were not. Therefore, a major research focus includes capturing and analyzing images of the entire surface of navel oranges in subsequent online detection work.

## Data availability statement

The raw data supporting the conclusions of this article will be made available by the authors, without undue reservation.

## Author contributions

JL: conceptualization, funding acquisition, writing – original draft review, and editing. ML: writing – original draft and visualization. LX: supervision and writing – review and editing. JC: conceptualization and funding acquisition. LH: conceptualization, investigation, software, and writing – review and editing. All authors contributed to the article and approved the submitted version.
